# Linezolid-Induced Lactic Acidosis Presenting As Acute Cholecystitis: A Case Report and Systematic Review

**DOI:** 10.7759/cureus.70794

**Published:** 2024-10-03

**Authors:** Fahima Akter, Hannah Bozell, Tyson Neumann, Cheng Chung

**Affiliations:** 1 Internal Medicine, Indiana University Health Ball Memorial Hospital, Muncie, USA; 2 Pediatrics, Albany Medical Center, Albany, USA; 3 Pulmonary and Critical Care Medicine, Indiana University Health Arnett Hospital, Lafayette, USA

**Keywords:** drug reaction, lactic acidosis, linezolid, oxazolidinones, zyvox

## Abstract

Linezolid has gained increased use for the treatment of infections caused by multidrug-resistant Gram-positive bacteria in recent years. It can cause rare but potentially life-threatening lactic acidosis. Here, we presented a case report of linezolid-induced lactic acidosis (LILA), along with a systematic review of current literature.

The patient was a 55-year-old male who presented with the symptoms of acute cholecystitis. He had been treated for sepsis due to acute cholecystitis with broad-spectrum antibiotics and intravenous fluids as per protocol. Still, his lactate level was getting elevated. After excluding other causes of lactic acidosis, LILA was diagnosed, and linezolid was discontinued. His lactic acid level, as well as his physical condition, improved after that.

Studies related to LILA were searched in Medline via PubMed. After screening titles, abstracts, and full texts, data were extracted, tabulated, and presented in this article. The risk of bias was also assessed. We found 78 relevant articles in the primary search, and 26 articles, including 496 patients, were included in the study. From 23 studies of 129 patients, 28 patients (21.7%) died in the setting of LILA. The peak lactate level in which the patient developed LILA was 38.1 mmol/L after four weeks of therapy. The most common health conditions associated with LILA were end-stage renal failure (ESRD), diabetes mellitus (DM), hypertension, chronic obstructive pulmonary disease (COPD), etc. Eighteen studies with a total of 30 patients discontinued it after the development of LILA. Twenty-four patients (80%) out of 30 survived after the discontinuation.

We recommend including LILA in the differential diagnoses when treating patients with lactic acidosis since LILA is associated with a relatively elevated mortality rate.

## Introduction

Linezolid is a drug of the oxazolidinone class, approved by the US Food and Drug Administration in 2000. It is mainly used for treating multidrug-resistant Gram-positive bacteria such as vancomycin-resistant *Enterococcus faecium*, vancomycin-resistant *Staphylococcus aureus*, multidrug-resistant tuberculosis, and methicillin-resistant *S. aureus*. Its use is increasing due to emerging orthopedic implant infections in the elderly and bone and joint infections [1,2].

Lactic acidosis can be classified into two types. Type A occurs due to hypoxia or decreased perfusion secondary to sepsis, hypovolemia, etc. Type B occurs due to deterioration of cellular metabolism secondary to other causes, such as drug toxicity, diabetes mellitus, alcoholism, malignancy, etc. [3]. Though the most common adverse effect of linezolid is reversible myelosuppression (anemia, thrombocytopenia, leukopenia), the first case of linezolid-induced lactic acidosis (LILA) was reported in 2003 [4,5]. It results in serious adverse effects such as multiorgan failure, which can be life-threatening. Little is known about the pathophysiology, risk factors, and characteristics of patients who develop LILA.

Here, we presented a case of a 55-year-old male with LILA whose symptoms mimicked acute cholecystitis, along with a review of current literature.

## Case presentation

**Figure 1:**
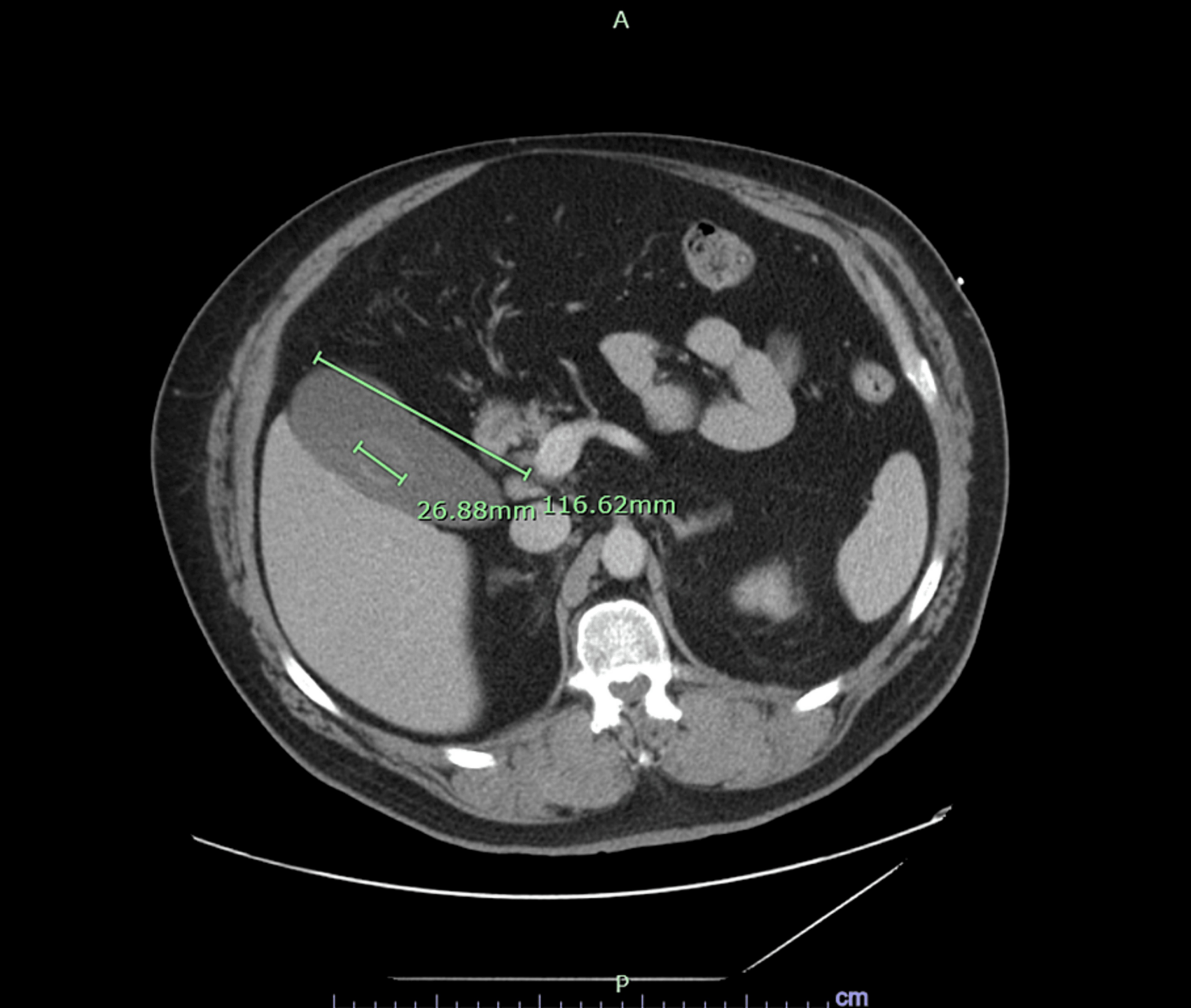
Enlarged Gallbladder (116.62 mm × 26.88 mm) in a Patient With Linezolid-Induced Lactic Acidosis

A 55-year-old male with a history of obesity, tobacco use, and uncontrolled diabetes mellitus complicated by osteomyelitis of the left foot presented to the emergency department with complaints of epigastric and left upper quadrant pain for one day associated with nausea and vomiting. Vitals signs were as follows: temperature of 35.5 degrees Celsius, heart rate of 123 bpm, blood pressure of 174/105 mmHg, and oxygen saturation of 97% on room air. Relevant labs were a WBC count of 14.3 k/cumm, lactate level of 7.3 mmol/L, pH 7.22, anion gap of 18 mmol/L, and creatinine of 1.39 mg/dL. No other abnormalities were noted on the rest of the complete metabolic panel. On a CT scan, the patient had an enlarged gall bladder (Figure 1).

Initially, the patient was diagnosed and treated for sepsis due to acute cholecystitis with broad-spectrum antibiotics and intravenous fluids per protocol. Despite an additional two-liter fluid bolus, the lactic acid level continued to be elevated.

On review of his medication history, the patient had a previous history of chronic diabetic left foot osteomyelitis and methicillin-sensitive *S. aureus* (MSSA) septicemia, requiring hospitalization for three days. He was treated with oral linezolid due to his inability to tolerate any adhesives required for a peripherally inserted central catheter (PICC) line. After six weeks of treatment with linezolid, the patient was presented to the emergency room.

After investigating alternative differentials such as hypoperfusion, tissue necrosis, or liver failure, LILA was suspected. Linezolid was discontinued and transitioned to doxycycline. After 12 hours of discontinuation, his lactate level decreased to 1.8 mmol/L, and his pH improved to 7.32. The laboratory findings during linezolid therapy and 12 hours after discontinuation are shown in Table 1.

**Table 1:** Time Course of Laboratory Test Results ALT: alanine transaminase; AST: aspartate aminotransferase; BUN: blood urea nitrogen; PO2: partial pressure of oxygen; PCO2: partial pressure of carbon dioxide; WBC: white blood cell

Pertinent Laboratory Values	Reference Range	Initial Findings	After 12 Hours of Discontinuation
pH Arterial	7.35-7.45	7.22	7.32
PCO2 Arterial (mmHg)	35-45	35	37
PO2 (mmHg)	80-110	77	68
Venous Lactate (mmol/L)	0.5-2.2	7.3	1.8
Anion Gap (mmol/L)	3-11	18	9
Glucose (mg/dL)	70-99	128	339
Alkaline Phos (Units/L)	25-125	47	-
ALT (Units/L)	7-52	44	-
AST (Units/L)	13-39	27	-
Bilirubin Total (mg/dL)	0.0-1.0	0.4	-
BUN (mg/dL)	5-20	27	18
Creatinine (mg/dL)	0.8-1.40	1.39	1.17
Sodium (mmol/L)	135-145	139	132
Potassium (mmol/L)	3.5-5.5	2.9	4.6
Chloride (mmol/L)	98-108	101	100
Bicarb (mmol/L)	22-29	15	18.9
WBC (k/cumm)	3.6-10.6	14.3	-

## Discussion

Methodology

We have done this systematic review, maintaining the standard of Preferred Reporting Items for Systematic Reviews and Meta-Analysis (PRISMA) guidelines, 2020 [6].

Eligibility Criteria

The inclusion criteria for this study encompassed all articles about LILA, written in English, and published studies, covering people of all ages, genders, ethnicities, and geographic locations. In contrast, the exclusion criteria ruled out editorials, short comments, letters to the editor, viewpoints, opinion papers, and commentary. Animal studies and articles without available full text were also excluded from the review.

Search Methodology

Following a comprehensive search criterion, we searched Medline through PubMed for studies of LILA until April 2023. The search strategy was developed following PICO criteria (Population, Intervention, Comparison, Outcome). Advanced searching was done following the keywords ("linezolid" [All Fields]) OR ("linezolid" [MeSH Terms]) OR ("zyvox" [All Fields]), ("lactic acidosis" [All Fields]) OR (lactic acidosis [MeSH Terms]) in combination.

Screening Process

Two reviewers individually screened the title and abstract of all eligible articles following the inclusion and exclusion criteria. After the primary title and abstract screening, relevant articles were selected for the full-text screening. Both reviewers independently reviewed all the selected articles. There were no duplicates. Any conflict of opinion that appeared in any screening steps is resolved by discussion of themselves and by the expert advice of the senior authors.

Data Extraction

All the articles have been read thoroughly for relevant information regarding LILA and were documented precisely. Data points including author name, study design, age and gender of the patients, comorbid conditions, duration, doses and route of linezolid, reasons for using this medication, if any concurrent medications used that can have an impact on lactate level, peak lactate levels, pH, therapeutic approaches, resultant effects, disease prevalence, and associated risk factors were reviewed and summarized.

Assessment of Risk of Bias

The risk of bias was assessed using the "JBI Checklist for Case Reports" [7], for case reports and case series and the RoBANS tool (Risk of Bias Assessment tool for Non-randomized Studies) for other observational studies [8]. Graphical representations of the risk of bias for case reports, case series, and observational studies are shown in two stacked bar charts.

Data Synthesis

A narrative data synthesis was performed to bring together the findings, examine the similarities and differences among the studies, and assess the strength of the evidence. Meta-analysis was not done because of the heterogeneity of including articles regarding interventions, outcomes, and scenarios to provide a meaningful summary.

Results

Characteristics of Included Studies

**Figure 2:**
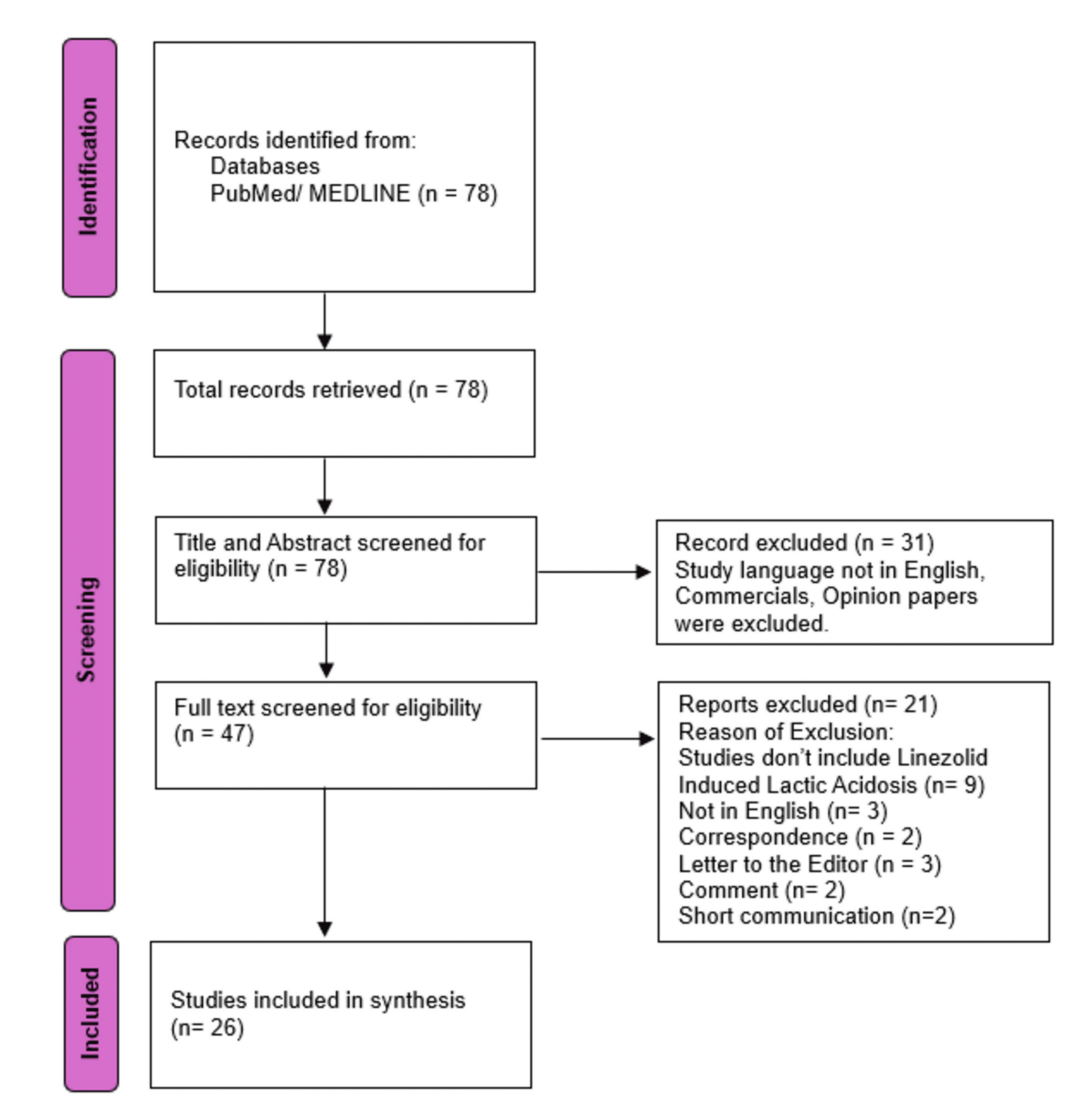
Flow Diagram of Identification of Studies

The study search and inclusion process are shown in the PRISMA flow diagram, 2020 (Figure 2).

In the initial search, 78 relevant articles were found. Forty-seven articles were selected for full-text screening after title-abstract screening. After full-text screening, 21 articles were excluded for not fulfilling our strict inclusion and exclusion criteria. Twenty-six articles with 496 patients were included in this study and selected for data extraction. Among 26 studies, 17 were case reports, two case series (including five cases total), two retrospective cohort studies, two cross-sectional studies, two reviews, and one case-control study. The characteristics and outcomes of the included studies are shown in Table 2.

**Table 2:** Characteristics of Included Studies and Treatment Outcomes AF: atrial fibrillation; AIDS: acquired immunodeficiency syndrome; AKI: acute kidney disease; ALL: acute lymphoblastic leukemia; AML: acute myeloblastic leukemia; AV: aortic valve; BMT: bone marrow transplant; CAD: coronary artery disease; CKD: chronic kidney disease; CLD: chronic liver disease; COPD: chronic obstructive pulmonary disease; CRRT: continuous renal replacement therapy; CVVH: continuous veno-venous hemofiltration; DM: diabetes mellitus; DVT: deep venous thrombosis; D50W: dextrose 50% in water; ESRD: end-stage renal disease; F: female; HD: hemodialysis; HF: heart failure; HTN: hypertension; IV: intravenous; M: male; MRSA: methicillin-resistant *Staphylococcus aureus*; MS: mitral stenosis; MV: mitral valve; NE: nor-epinephrine; NR: not-reported; PH: portal hypertension; PVD: peripheral vascular disease; qm: every month; qn: every night; q/12h: every 12 hours; SC: subcutaneous; UTI: urinary tract infection; VRE: vancomycin-resistant *Enterococcus faecium*

S. no.	Author name	Study design	Age (years)	Sex	Comorbid conditions	Reason of therapy	Concurrent medications	Duration of therapy	Dose and route	Peak lactate (mmol/L)	pH	Treatment	Outcome
1	Narita et al. [9]	Minireview	64.8	F=4; M=2	AML, BMT Biliary cirrhosis, HTN, Cutaneous T-cell lymphoma	Prosthetic joint infection, Osteomyelitis, Disseminated Nocardiosis	NR	1-16 weeks	NR	10-24.5	NR	Discontinued	Survived (n=5); Died (n=2)
2	Ozkaya-Parlakay et al. [10]	Cross-sectional study	Mean= 6.1 years	F=20 M=30	NR	Bacteremia, Pneumonia, UTI, Sepsis,	Amikacin, Ciprofloxacin, Caspofungin, Ceftriaxone, Imipenem, Clarithromycin, Fluconazole, Ornidazole, Ampicillin/sulbactam, Vancomycin, Trimethoprim/sulfamethoxazole, Clindamycin, Amphotericin B	18.1 (3-93 days)	10 mg/kg, q/8h in age 0-11 years, 10 mg/kg q/12h in older children.	NR	NR	NR	Nine children died
3	Smolka et al. [11]	Case report	9y	F	B-cell-precursor ALL, Allogeneic hematopoietic stem cell transplant	Multifocal pulmonary abscesses by mycobacterium	Amikacin, Meropenem, Posaconazole, Valaciclovir, Prednisone, Budesonide	51 days	810 mg/day	19	7.06	Discontinued, Sodium bicarbonate	Survived
4	Su et al. [12]	Case series (Case-1)	6 months	M	Prematurity, Necrotizing enterocolitis, Liver disease with coagulopathy, Bronchopulmonary dysplasia,	Surgical site MRSA following repair of mucocutaneous fistulas, VRE bacteremia	NR	39 days	NR	24	NR	Discontinued	Died
	Su et al. [12]	Case-2	6 months	F	Protein-losing enteropathy, Hepatic insufficiency	Prior VRE infection, Suspected sepsis	NR	4 weeks	NR	38.1	NR	NR	Died
	Su et al. [12]	Case-3	16 Y	M	Cryptogenic cirrhosis, Bilateral hepatic vein occlusion, PH	VRE in urine	NR	7 days	NR	28	NR	CRRT	Died
5	Liu et al. [13]	Case-control study	94	M+F=35	CKD (n=24), HTN (n=29), AF (n=16), CAD (n=29), DM (n=19), COPD (n=27)	Pulmonary, skin, and soft tissue infections	NR	10 days	IV 600 mg qm, Oral 600 mg qn	4.6	NR	NR	30-day mortality rate = 48.6%
6	Mori et al. [14]	Retrospective cohort study	>20 yrs	M=65 F=29	DM (n=19), COPD (n=10), CKD (n=13), HF (n=11), CLD (n=5), malignancy (n=29),	Respiratory Tract Infection	NR	8-10 days	NR	17.1% developed lactate >5	<7.35	NR	30 days mortality 28.8%
7	Im et al. [15]	Retrospective Cohort study	61.4± 17	F=2 M=3	DM (n=4), CKD (n=2), AKI (n=2)	Skin and soft tissue infection, Bacteremia, Pneumonia, Intra-abdominal infection	NR	19.7± 18 days.	NR	>4	<7.25	HD (n=2), Bivone (n=1), none (n=1)	Survived (n=3), Died (n=2)
8	Velez and Janech [16]	Case Report	36 y	Male	ESRD	VRE bacteremia	Meropenem, Metronidazole Gabapentin, Metoprolol, Clonidine, Pantoprazole, Hydrocodone, Darbepoetin, Metoclopramide	6 weeks	600 mg q/12h	12.5	7.31	Discontinued	Survived
9	Del Pozo et al. [17]	Case Series (Case-1)	72y	F	DM, Liver transplant due to primary biliary cirrhosis,	Disseminated nocardiosis (i.e., bone and central nervous system)	Ceftriaxone	9 weeks	600 mg q/12h	4.8	7.25	Discontinued, Thiamine	Survived
	Del Pozo et al. [17]	Case-2	43y	M	Liver transplant, Acute renal failure	Lung tuberculosis	Ethambutol, Isoniazid	8 weeks	600 mg q/12h	7.20	7.29	Discontinued, Bicarbonate, Thiamine	Survived
10	Nightingale et al. [18]	Case report	67 y	M	COPD, CAD, HTN, CKD	Post-operative intra-abdominal sepsis, Pneumonia, bacteremia after elective abdominal aortic aneurysm repair	NR	7 days	600mg q/12h	7.7	NR	Discontinued	Survived
11	Kopterides et al. [19]	Case Report	70y	M	cutaneous T-cell lymphoma,	MRSA bacteremia	NR	7 days	NR	12.5	NR	Discontinued, thiamine 100 mg per day	Survived
12	Santini et al. [20]	Review	63±17	F=22 M=26	Renal Failure (n=13)	NR	NR	35±29 days	600mg, q/12h	13±7	7.14± 0.16	NR	Survived (n=39), Died(n=9)
13	Mao et al. [21]	Case Report	57	M	T-2 DM, Poor renal function	Severe Pneumonia	IV Imipenem, Cilastatin sodium, Vancomycin	NR	600 mg, IV q/12h, 3 doses	10	NR	Discontinued	Died
14	Dai et al. [22]	Cross-sectional study	18-60	M =125 F=111	HTN, DM	Infectious diseases,	Meropenem, Warfarin, Ertapenem	1-2 weeks	IV, Oral	NR	NR	NR	NR
15	Xiao et al. [23]	Case Report	50	F	AV, MV replacement, Rheumatic heart disease	Endocarditis	Warfarin	25 days	600 mg IV q/12h	19	6.94	HD, NE	died
16	Wiener et al. [24]	Case Report	80	F	MS	Post-operative bacteremia after MV replacement	Amiodarone, Metoprolol, Pantoprazole, Coumadin.	19 days	IV, Oral	19	7.026	Discontinued	Survived
17	Tobias et al. [25]	Case report	52 y	F	HTN, ulcerative colitis, osteoarthritis	Diverticulitis complicated by abscess	NR	6 weeks	600 mg IV piggyback (IVPB) q/12 h	16.1	7.187	Thiamine 100 mg IV push daily, HD	Survived
18	Zuccarini et al. [26]	Case report	67y	F	HTN, dyslipidemia, bilateral avascular necrosis of the femoral head	Post-operative MRSA infection after left total hip replacement	NR	6 weeks	NR	9.4	NR	Discontinued	Survived
19	Chen et al. [27]	Case report	54y	M	Lupus nephritis, Uremic state, Cadaveric kidney transplant	Pulmonary non-tuberculosis mycobacterial infection	Azithromycin, Prednisolone, Mycophenolic acid, Sirolimus	NR	600 mg q/12h	18.4	7.122	HD for 4 hours	Survived
20	Protti et al. [28]	Case Report	64 y	M	Single lung transplant, Pulmonary fibrosis,	Acute respiratory failure due to ground glass opacities at the right lung (graft)	Meropenem, Amphotericin-B, Methylprednisolone	NR	1200 mg/day IV continuous infusion	22	NR	Discontinued	Died
21	Johnson et al. [29]	Case Report	34 y	M	Sickle cell disease, renal disease, Secondary hemochromatosis, Stroke, seizure	VRE bacteremia	Amphotericin B, Micafungin, Piperacillin/Tazobactam	11 days	600 mg IV q/12h	26	7.07	Discontinued, several amps. of D50W	Survived
22	Belani et al. [30]	Case Report	81y	F	PVD, CAD	Deep heel abscess by VRE	IV Meropenem	12 days	NR	14.2	6.89	Discontinued	Survived
23	Hsu et al. [31]	Case Report	38y	F	HTN, alcoholic cirrhosis, stage 3 CKD	VRE UTI	NR	12 days	NR	24	6.918	Discontinued, Continuous HD, IV Thiamine	Survived
24	Carson et al. [32]	Case Report	35 y	F	AIDS	Disseminated infection with *Mycobacterium avium*-intracellular complex	Moxifloxacin, Tenofovir, Emtricitabine, Efavirenz, Pyridoxine, Ethambutol, Clarithromycin, Atovaquone, Valacyclovir, Erythropoietin, Sargramostim, Leucovorin	35 days	600 mg q/12h	11.38	7.16	Discontinued, CVVH, Riboflavin, Thiamine, Folic acid, Coenzyme Q10, L-carnitine	Survived
25	Scotton et al. [33]	Case Report	81y	F	NR	Bacterial spondylodiscitis, Disseminated disease with involvement of the meninges, Mediastinum lymph nodes, Bilateral pleural effusion, Paravertebral abscess	Isoniazid Rifampin, Ethambutol	12 days	600 mg q/12h	18.6	7.25	Discontinued, Bicarbonate infusion	Survived
26	Lee et al. [34]	Case report	56y	Caucasian F	Renal transplant, Gastric bypass for morbid obesity,	Urosepsis	Doxycycline Liposomal amphotericin- B, Meropenem	3 days	600 mg q/12h	4.3	7.19	Discontinued,	Survived

Evidence of LILA

The lowest duration of linezolid therapy that caused lactic acidosis was three days for a 56-year-old Caucasian female due to urosepsis with a peak lactate level of 4.3 mmol/L. Her condition improved after discontinuation of the therapy [35]. One mini-review including six patients revealed that the highest duration of therapy was 112 days (16 weeks) with a peak lactate level of 24.5 mmol/L [9].

Ninety-six patients from 14 studies who developed lactic acidosis received a standard dose of linezolid (600 mg twice a day). One cross-sectional study included 50 patients receiving linezolid 10 mg/kg three times daily for 0-11 months old and 10 mg/kg twice daily for older children [10]. One nine-year-old female patient received 810 mg/day for 51 days before emerging LILA [11]. Ten studies did not mention the dosing of linezolid.

Among these 26 studies, the peak lactate level associated with LILA was 38.1 mmol/L, developed in a six-month-old child after four weeks of linezolid therapy [12]. Three studies did not specifically mention peak lactate levels.

From 23 studies, including 129 patients, 28 (21.7%) patients died in the setting of LILA. Three studies were not included for lack of mortality data. One case-control study in people older than 85 years showed that 30-day mortality was 48.6%, even after a relatively shorter duration of linezolid therapy. The risk factors associated with lactic acidosis development after linezolid use in patients older than 85 are the duration of treatment longer than nine days (odds ratio (OR): 3.541; 95% confidence interval (CI): 1.161-10.793; p = 0.026), an arterial blood glucose level >8 mmol/L (OR: 4.548; 95% CI: 1.507-13.725; p = 0.007), and a high sequential organ failure assessment score (OR: 1.429; 95% CI: 1.213-1.685; p < 0.0001) [13]. Another study showed that they found 28.8% mortality after 8-10 days of administration of linezolid in 94 patients older than 20 years [14].

Out of 496 individuals, 261 were male and 200 were female. The gender of 35 patients was not specified. Regarding age, 369 individuals were under 65, while 102 were over 65. The age of 25 participants was not provided.

Risk of Bias Assessment of the Included Studies

**Figure 3:**
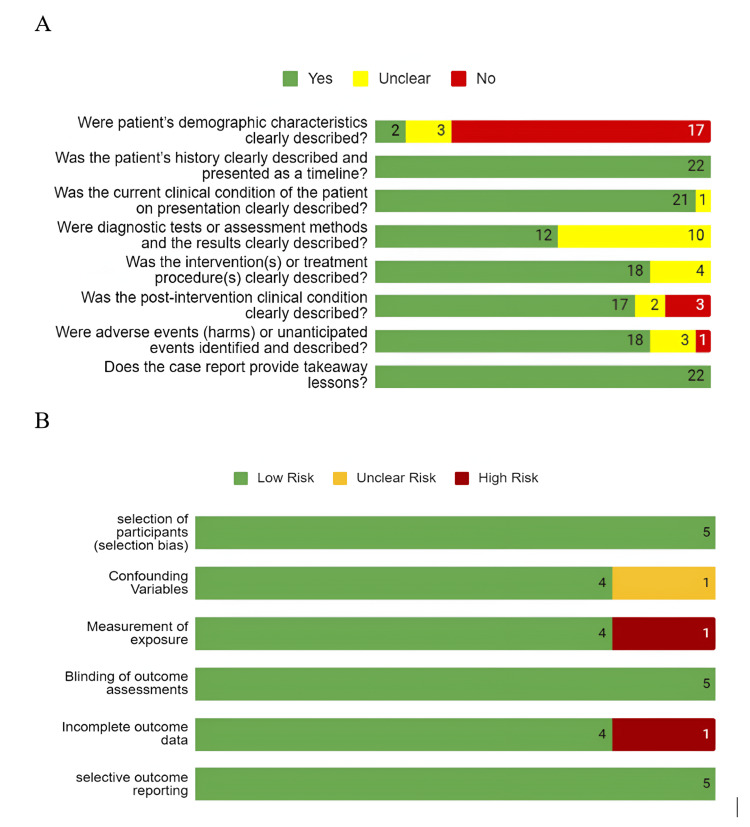
Risk of Bias of the Included Studies (n = number of articles) (A) Risk of Bias of the Case Reports and Case Series; (B) Risk of Bias of the Non-randomized Studies

We assessed the risk of bias for 24 articles, as two out of 26 articles were reviews. The detailed findings are shown in Figure 3.

"JBI Checklist for Case Reports" was used to analyze the bias of all case reports and case series. Eight domains of this tool, including demographic characteristics, history, current clinical condition, diagnostics tests, interventions and treatment procedure, post-intervention clinical condition, adverse events, and takeaway lessons, were checked precisely and noted.

For observational studies, we checked six domains of the RoBANS tool, including participant selection, confounding variables, measurement of exposure, blinding of the outcome assessments, incomplete outcome data, and selective outcome reporting.

Among the 22 case reports, 17 didn't describe patients' demographic location clearly. Most case reports described patients' history (n=22) and clinical condition on presentation (n=21). Ten studies didn't mention assessment methods and results, i.e., the dose of linezolid administration, lactic acid level after discontinuation of linezolid, didn't exclude the differential diagnosis of lactic acidosis like sepsis, etc. Seventeen case reports described properly the post-intervention clinical condition of the patients.

For observational studies, we assessed the six domains of the RoBANS tool. Among the six studies, none had selection bias, blinding of the outcome assessments bias, or selective outcome reporting bias. One study didn't mention if they assessed the patient for potential confounding bias. One study had a high risk of measurement of exposure bias as the data was self-reported by the patient, which can cause recall bias. One study mentioned that they had missing data. Hence, the incomplete outcome bias risk is high.

Discussion

Lactic acidosis is a condition with serum lactate level >4 mmol/L and metabolic acidosis (pH <7.35) in arterial blood gas analysis [13]. Common risk factors for LILA are pregnancy, renal impairment, obesity, lipid dystrophy, female sex, etc. [15].

Although in our study we found a larger number of males (260) were affected compared to females (200), the US Food and Drug Administration reported 90 LILA cases from August 2011 to August 2016 [36]. Since 2003, it has been thought that prolonged use of linezolid leads to lactic acidosis due to mitochondrial toxicity [4]. Though a standard dose of 600 mg twice a day is thought to be non-toxic, evidence suggests that advanced age, impaired renal function, prolonged therapy, and co-administration of some drugs, e.g., omeprazole, amiodarone or amlodipine increase the risk of lactic acidosis due to mitochondrial toxicity [1,37-39].

Linezolid most likely binds to the A-site of the peptidyl transferase center in the 23rRNA of the bacterial ribosome and disrupts the correct positioning of aminoacyl-tRNAs. Similarly, they can bind to the human mitochondrial ribosomes at the same location in the 16S rRNA and interfere with protein synthesis. It explains some of the effects of lactic acidosis associated with linezolid [40-42].

De Vriese et al. demonstrated that linezolid can inhibit protein synthesis in human and animal tissue after prolonged use. Though mitochondrial morphology didn't change, biochemical analysis revealed severe inhibition of respiratory chain complex I in the muscle and kidney and inhibition of complex IV in the muscle, kidney, and liver in humans. Those complexes are encoded by mtRNA. No change was noticed in complex II, which is encoded by nuclear DNA. Those findings were also detected in rats. mtRNA-encoded respiratory complexes were severely reduced after high doses and prolonged administration of linezolid [43].

Garrabou et al. also found decreased complex 4 activity and cytochrome c oxidase subunit 2 level in peripheral blood mononuclear cells of patients with LILA, which resolved with drug withdrawal [44].

In our study, we have found 496 cases of LILA. Patients mainly received this treatment due to pneumonia, MRSA bacteremia, urinary tract infection (UTI), sepsis, skin and soft tissue infection, pulmonary mycobacterial infection, disseminated *Mycobacterium avium* complex (MAC) infection, disseminated nocardiosis, diverticulitis, endocarditis, etc.

Previous studies denoted that LILA develops after prolonged use of linezolid [4,16,45-46]. A case series of two patients, 72- and 43-years old, male and female, received linezolid for nine and eight weeks, respectively, before developing lactic acidosis [17]. But LILA is also reported to occur even after a short duration of therapy of three to seven days. Their age ranged from 16 to 70 years, which supports that LILA can develop in patients of any age with an even shorter duration of medication use [12,18,19,35].

The majority of patients had underlying health conditions. These included prevalent conditions such as end-stage renal failure (ESRD), diabetes mellitus, hypertension, COPD, coronary artery disease, chronic liver disease, as well as a history of kidney and liver transplantation.

Linezolid is primarily metabolized by the liver, with the remaining 30% metabolized by the kidney. Kidney excretion occurs when the lactic acid level is above 6-10 mmol/L [47]. Therefore, severe renal impairment can be a risk factor for lactic acidosis [16]. In our study, a total number of 57 patients had renal impairment. Three separate studies of 24, 13, and another 13 patients with renal failure and CKD developed lactic acidosis after linezolid use. Mori et al. showed that renal insufficiency (estimated glomerular filtration rate (eGFR)) <30 mL/min (OR: 7.4; 95% CI: 1.0-84.4, p = 0.02) increases the risk of LILA [14].

Seventeen patients either had chronic liver disease, liver transplants, or biliary cirrhosis. Five out of 12 patients didn't survive even after discontinuation of the drug or continuous renal replacement therapy (CRRT). One study of 94 patients in which five of them had chronic liver disease denoted that their overall 30-day mortality was 28.8% [11]. A six-month-old female patient with hepatic insufficiency and protein-losing enteropathy developed LILA with a peak lactate level of 38.1 mmol/L after receiving four weeks of linezolid. Although the study did not mention the dosing and treatment, the patient unfortunately did not survive [12].

According to Jae et al., diabetes is familiar to be a cause of lactic acidosis, but no statistically significant differences between diabetes and LILA were found [16]. Forty-five patients (9.07%) out of 496 had diabetes in this study. Further studies on this subject need to be carried out to know if diabetes is the definitive risk factor for LILA.

The treatment is discontinuation of the medication. Twenty-three studies from 21 articles discussed the treatment modalities used for LILA. Seventeen articles, including 18 studies with 30 patients, denoted that they discontinued linezolid after developing lactic acidosis. Twenty-four patients out of those 30 survived after the discontinuation. The rest of the studies did not mention the treatment modality or the survival status.

Five patients required hemodialysis. The only death was a 16-year-old male with vancomycin-resistant *Enterococcus faecium* UTI requiring CRRT [13]. Some patients also received thiamine and sodium bicarbonate with unclear efficacy.

Our study has several limitations. Due to the relatively small sample size, a definitive conclusion cannot be made. Therefore, more relevant studies are necessary for more substantial evidence. Due to data heterogenicity, meta-analysis was not possible. We could not account for all the confounding factors, such as disease severity, use of other unreported medication, etc. All 26 studies were either observational studies or reviews. Among them, 17 were case reports, three cross-sectional studies, two case series, two reviews, one case-control, and one retrospective cohort study.

## Conclusions

Linezolid use is associated with lactic acidosis with a relatively elevated mortality rate. We recommend including LILA in the differential diagnoses when treating patients with lactic acidosis.
